# Fidelity of the Guided Self-Determination program in the MyHealth study during breast cancer follow-up

**DOI:** 10.2340/1651-226X.2025.42253

**Published:** 2025-02-17

**Authors:** Sigrid N. Biener, Beverley L. Høeg, Lena Saltbæk, Susanne O. Dalton, Christoffer Johansen, Randi V. Karlsen, Federica Belmonte, Vibeke Zoffmann, Pernille E. Bidstrup

**Affiliations:** a Psychological Aspects of Cancer, Danish Cancer Institute, Copenhagen, Denmark; b Department of Nutrition, Exercise and Sports, University of Copenhagen, Copenhagen, Denmark; c Department of Clinical Oncology, Zealand University Hospital, Næstved, Denmark; d Cancer Survivorship, Danish Cancer Institute, Copenhagen, Denmark; e Institute of Clinical Medicine, Faculty of Health, Copenhagen University, Copenhagen, Denmark; f Statistics and Data Analysis, Danish Cancer Institute, Copenhagen, Denmark; g Department of Epidemiology Research, Statens Serum Institut, Copenhagen, Denmark; h Department of Public Health, University of Copenhagen, Copenhagen, Denmark

**Keywords:** Breast cancer, nurse-led, follow-up, fidelity, acceptance

## Abstract

**Background and purpose:**

MyHealth is a new follow-up program including individual nurse-led sessions based on Guided Self-Determination (GSD), which has been shown to improve health and psychological outcomes in patients after treatment for breast cancer. Fidelity assessment is important to support the implementation of GSD in clinical practice. The purpose of this study was thus to investigate fidelity and acceptance of the GSD program in the MyHealth study and whether sociodemographic and psychological factors were associated with patients’ completion of the GSD program and completion of reflection sheets.

**Material and methods:**

We assessed fidelity quantitatively by examining patients’ completion of the GSD program (i.e. ≥3 sessions), completion of the reflection sheets and their associations with sociodemographic and psychological factors among 239 patients, and nurse-reported acceptance qualitatively through a focus group interview with all five nurses providing the GSD program.

**Results:**

A total of 81% of patients completed the GSD program, while 71% of the reflection sheets were completed. Including a relative in a GSD session and lower education were significantly associated with completion of the program. Younger age and including a relative in a GSD session were significantly associated with completion of reflection sheets. Nurses found GSD highly applicable and especially appreciated a values-clarifying GSD reflection sheet and the inclusion of a relative.

**Interpretation:**

The GSD program was applied with moderate-to-high fidelity, and the inclusion of relatives is potentially valuable. The GSD program indicates high usability and potential for being translated into clinical practice.

## Introduction

Traditionally, follow-up after treatment for breast cancer (BC) has focused primarily on the detection of recurrence and new primary cancer disease with less attention to symptom management and psychological care [1–3]. However, a systematic review on BC follow-up programs found that patients with BC report unmet needs throughout the cancer trajectory, proposing a need to strengthen physical and psychological support and self-management skills [4]. Additionally, there is uncertain evidence for the effectiveness of traditional specialist-led follow-up compared to non-specialist-led follow-up across cancer sites, underlining the potential for non-specialist healthcare providers (HCPs), such as nurses leading the follow-up care while maintaining the same quality of care [5].

MyHealth is a nurse-led follow-up program based on Guided Self-Determination (GSD) and use of patient reported outcomes (PROs) [1]. GSD is an evidence-based method to guide patients and HCPs through a process of shared decision-making and mutual problem solving with the use of semi-structured reflection sheets [6]. GSD has been shown to improve long-term illness management in patients with gynecological cancer and diabetes [6, 7] and is a standard care in various clinical settings [8, 9]. A recent randomized controlled trial (RCT) showed that patients in the MyHealth intervention group had significantly higher BC-specific quality of life (QoL), less fear of cancer recurrence (FCR), and symptoms of anxiety and depression through 3 years of follow-up compared to patients in the control group with traditional physician-led follow-up [10]. In order to translate these positive results into clinical practice, it is important to assess fidelity, here defined as the degree to which the intervention is implemented as intended and acceptance including HCPs experience with the intervention being applicable, valuable, and worth implementing [11–13].

Previous studies have examined factors related to fidelity to treatment programs, including patient sociodemographic factors, physical and mental health, and clinical characteristics [14–18]. However, to our knowledge, no studies have systematically investigated factors related to the fidelity of a GSD-based program.

The aims of this study were to investigate fidelity and acceptance of the GSD program as part of the MyHealth study. Fidelity was assessed quantitatively as completion of the GSD program (i.e. ≥3 sessions) and completion of the reflection sheets, and whether sociodemographic and psychological factors were associated with these. Acceptance was assessed qualitatively based on the nurses’ judgment of the applicability and value of the reflection sheets.

## Materials and methods

### Study design

This study was designed in accordance with fidelity assessment methods [19] as a multi-methods evaluation of the GSD program in the MyHealth study [1, 10] using both quantitative and qualitative data.

### MyHealth study

The MyHealth study is as previously described [1, 10] a two-armed 1:1 RCT (ClinicalTrials.gov identifier:NCT02949167). Patients were invited if they fulfilled the criteria: completed primary treatment at the Zealand University Hospital, Denmark, for early BC (stage I–II) between January 2017 and January 2019 without clinical signs of recurrent disease, age >40 years at diagnosis, performance status ≤3 by the Eastern Cooperative Oncology group scale, sufficient proficiency in Danish, and provided written informed consent [1, 10]. Exclusion criteria were as follows: clinical sign of residual disease, genetic predisposition for BC, recurrent cancer, the presence of other active cancers except non-melanoma skin cancer, severe cognitive problem, and severe psychiatric disease or alcohol abuse or narcotic dependence [10]. The intervention group received a follow-up program consisting of 3–5 individual GSD sessions per patient preference with an experienced oncology nurse during the first 6 months. Additionally, the follow-up program included repeated reporting of symptoms using PRO and support in symptom management and patient navigation through 3 years of follow-up. PRO included symptoms of recurrence, adverse effects, and late effects of BC treatment collected through digital or paper questionnaire at time points 0, 3, 6, 9, 12, 18, 24, 30, and 36 months following inclusion [1, 10]. The GSD sessions 1, 2, and 5 were mandatory and defined as the *short version* of the program, whereas sessions 3 and 4 were elective, and all five sessions were defined as the *long version* of the program. Patients were encouraged to invite a relative in the second session [1, 10]. The control group received follow-up consultations with a physician every 6 months for 3 years. Thereafter, patients in both groups received the standard regional follow-up program from year 3 to year 10. The primary outcome in the MyHealth study was QoL, while secondary outcomes included FCR, anxiety, depression, time to detection of recurrence, and use of healthcare services through 5 years of follow-up.

### Guided Self-Determination

GSD aims to support patients developing life skills in managing their disease by guiding patients and HCPs through mutual reflection supported by semi-structured reflection sheets [6, 20] and was adjusted here to patients treated for BC. An overview of the total 21 GSD reflection sheets is shown in Supplemental Table 1. Five nurses with 10–18 years of experience in oncology attended a comprehensive 6-day GSD training supervised by VZ and thereafter provided the GSD sessions, monitored symptoms registered in PRO, instructed on self-management, and referred to the project physician (LS) at symptoms suspicious to recurrence.

### Study population and procedure

251 participants were assigned to the MyHealth intervention group, of which 12 participants were excluded due to exclusion criteria (withdrawal of consent, genetic predisposition, recurrence, and other cancer), leaving 239 participants in the current study [10].

*Completion of the program* was defined as participating in at least the short version of the GSD program, determined by the completion of one corresponding reflection sheet for sessions 1, 2, and 5 as a minimum. *Completion of reflection sheets* was defined as the number of reflection sheets in the *short version (maximum 13 sheets)*. Patients were encourage to complete the reflection sheets in writing except for the ‘Invitation to collaborate’ which was presented verbally by the nurses. Fidelity cut-off values applicable for both completion of the program and completion of reflection sheets have previously been established: ≥80% indicates high fidelity, 51–79% indicates moderate fidelity, and ≤50% indicates low fidelity [11].

We used baseline questionnaire data from all 239 patients on sociodemographic (age, education, and cohabitation status) and psychological (QoL, symptoms of depression, symptoms of anxiety, patient activation, and FCR) factors.

Sociodemographic factors were measured as age (≥60; <60 years), education (basic or high school; vocational training; higher education), and cohabitation status (living with a partner; living alone). QoL (continuous) was measured with the Functional Assessment of Cancer Therapy-Breast (range 0–148) with higher scores reflecting better QoL [21]. Symptoms of depression (continuous) were measured with the Patient Health Questionnaire-9 with higher scores indicating increased severity (score ≥ 10 indicating moderate to severe depression) [22]. Symptoms of anxiety (continuous) were measured with the Generalized Anxiety Disorder-7 with higher scores indicating increased severity (score ≥ 10 indicating generalized anxiety) [23]. Patient activation (continuous) was measured with the Patient Activation Measure-13 (range 0–100) with higher scores indicating higher confidence in ability to manage disease [24]. FCR (continuous) was measured with the concerns about Recurrence Questionnaire-4 with higher scores indicating increasing FCR (score ≥ 12 indicating clinical fear of recurrence) [25]. Information on inclusion of a relative (yes; no) was based on information registered by the nurses.

### Statistical analyses

Fidelity of the GSD program was investigated as frequencies and percentages of the patients’ completion status and completion of reflection sheets. Logistic regression analyses were applied to examine associations between characteristics on age, education, cohabitation status, inclusion of a relative, QoL, symptoms of depression, symptoms of anxiety, patient activation, and FCR and whether patients completed the GSD program. In linear regression models, we examined whether age, education, cohabitation status, QoL, symptoms of depression, symptoms of anxiety, patient activation, FCR, and inclusion of a relative were associated with the patients’ total number of completed reflection sheets. Assumptions for linear models were tested using visual plots, Kolmogorov-Smirnov Tests, and Levene’s Test for Equality of Variance. In analyses on including a relative, we excluded the reflection sheet ‘Partner’s unfinished sentences’ as this was intended for relatives to fill out. Supplementary analyses were further adjusted for age and education as these factors were considered potential confounders [26]. Statistical analyses were performed using SPSS Statistics 27.

### Focus group interview

Two experienced qualitative researchers (SNB and VZ) conducted a semi-structured focus group interview with the five nurses in September 2020 and thus within 15 months of the intervention ended. The purpose was to achieve knowledge on the nurses’ acceptance, thus their experiences with providing the GSD sessions and their perspectives on completion. The nurses were asked to recall three experiences with the GSD program including one positive and one challenging experience, and one case where a patient did or did not include a relative. Sharing experiences was used as a method to initiate the discussion [27]. The nurses were also asked independently to rank the three most valuable reflection sheets, and the reflection sheets they thought could be omitted. The focus group interview was audio-recorded and transcribed verbatim.

### Thematic analysis

Acceptance of the GSD program was investigated using thematic network analysis. We coded the material, identified themes, and constructed networks using Jennifer Attride-Stirling’s step-by-step guide [28]. The coding process was driven by identifying factors affecting patients’ completion of the GSD program, while still being open to new and changing categories. Abstractions from coded text segments were arranged into similar coherent groupings constructing thematic networks as a simple way to structure and organize themes at different levels to facilitate deeper levels of understanding [28].

## Results

In total, 239 patients were included in this study, the mean age at baseline was 59.9 years (9 SD), and 18% had basic or high school as the highest education. Most of the patients were living with a partner (69%), and most patients included a relative at the second GSD session (67%). The mean level of QoL was 107.2 reflecting high QoL, while the mean level of FCR was 14.6, which is above the cut-off score for clinical fear of recurrence (Table 1).

**Table 1:** Patient characteristics at baseline of 239 patients in the MyHealth intervention group.

Characteristics	Total frequency *n* (%)
**Age**	
≤ 60 y	136 (57)
> 60 y	103 (43)
Education^a^*	
Basic or high school	42 (18)
Vocational training	35 (15)
Higher education	161 (68)
**Cohabitation status**	
Living with a partner	166 (69)
Living alone	73 (31)
**Inclusion of a relative**	
Yes	161 (67)
No	78 (33)

	Mean (SD)

**Age**	59.9 (9)
**QoL**	107.2 (20)
**Symptoms of depression** ^a^	6.1 (5)
**Symptoms of anxiety^a^**	4.2 (4)
**Patient activation^b^**	65.1 (15)
**FCR^a^**	14.6 (10)

QoL: quality of life; FCR: fear of cancer recurrence; SD: standard deviation.

a Missing *n* = 1,

b Missing *n* = 16.

* Percentages may not sum to 100% due to rounding.

*n* = 239.

### Completion of the GSD program and completion of reflection sheets

A total of 81% completed the GSD program, and of these, 74% completed the short version and 26% the long version. The patients completed on average 71% of the reflection sheets of the *short version*. The three most frequently completed reflection sheets were ‘Invitation to collaborate’ presented verbally by nurses to all patients, ‘Symptoms to pay attention to after breast cancer treatment’ completed by 95% of the patients, and ‘Unfinished sentences’ completed by 90% of the patients. Session two included the reflection sheet ‘Partner’s unfinished sentences’ completed by the relative of 67% of the patients (Table 2).

**Table 2:** Reflection sheets completed by 239 patients in the MyHealth intervention group.

Reflection sheet	Completed *n* (%)
1.a Invitation to collaborate	239 (100)
1.b Important events and periods in your life	194 (81)
1.c What do you find difficult at present?	211 (88)
1.d.1 + 2 Unfinished sentences	215 (90)
1.e A picture, metaphor, or expression describing your life	150 (63)
1.f Current experiences on difficulties and nuisances	203 (85)
2.a Partner’s unfinished sentences	161 (67)
2.b My daily life and needs for changes	182 (76)
2.c Room for the fact, that you have been treated for cancer	181 (76)
3.a Current problem-solving	71 (30)
3.a.1 Your draft	61 (26)
3.a.2 The nurse’s draft	60 (25)
4.a.1 Your observations	50 (21)
4.a.2 Your thoughts and feelings	50 (21)
4.a.3 Your goals and intentions	49 (21)
4.a.4 Your actions^a^	47 (20)
4.b Dynamic problem solving	43 (18)
5.a Symptoms to pay attention to after breast cancer treatment	228 (95)
5.b New strategies and long-term plan for change^b^	115 (49)
5.c Pros and cons^b^	61 (26)
5.d Bullet points you want passed on^b^	62 (26)

a Missing *n* = 1,

b Missing *n* = 2.

*n* = 239.

### Factors influencing patients’ completion of the GSD program and completion of reflection sheets

In adjusted analyses, including a relative and lower education were associated with completing the program. Patients not including a relative had 96% lower odds (OR [odds ratio] 0.04 CI [confidence interval] [0.02;0.09]) of completing the program compared to patient including a relative, and patients with higher education had 73% lower odds (OR 0.27 CI [0.08;0.095]) of completing the program compared to patients with basic or high school (Table 3). Including a relative and age were associated with the total number of completed reflection sheets. Patients not including a relative completed on average 3.82 fewer sheets (β -3.82 CI [-5.02; -2.62]) compared to patients including a relative. Patients above 60 years completed on average 1.89 fewer sheets (β -1.89 CI [-3.18; -0.60]) compared to patients below age 60 (Table 4). Cohabitation status, QoL, symptoms of depression and anxiety, patient activation, and FCR were significantly associated with neither the completion of the program nor the completion of reflection sheets. In some cases, the assumptions of normality for the linear models were not met due to floor effects.

**Table 3:** Associations between patient characteristics, symptoms, and completion of the GSD program among 239 patients in the MyHealth intervention group.

Characteristics	Unadjusted		Adjusted^a^	
	OR [95% CI]	*P*	OR [95% CI]	*P*
**Age**				
≤ 60 y	1.00	-	1.00	-
> 60 y	0.94 [0.49; 1.80]	0.84	0.83 [0.42; 1.64]	0.60
**Education**				
Basic or high school	1.00	-	1.00	-
Vocational training	0.31 [0.07; 1.30]	0.11	0.29 [0.07; 1.24]	0.10
Higher education	0.29 [0.08; 0.99]	0.05*	0.27 [0.08; 0.95]	0.04*
**Cohabitation status**				
Living with a partner	1.00	-	1.00	-
Living alone	0.67 [0.34; 1.32]	0.24	0.67 [0.33; 1.33]	0.25
**Inclusion of a relative^b^**				
Yes	1.00	-	1.00	-
No	0.04 [0.02; 0.09]	< 0.01*	0.04 [0.02; 0.09]	< 0.01*
**QoL**	1.00 [0.98; 1.01]	0.59	1.00 [0.98; 1.02]	0.85
**Symptoms of depression**	0.97 [0.91; 1.03]	0.29	0.96 [0.89; 1.02]	0.17
**Symptoms of anxiety**	1.02 [0.94; 1.10]	0.71	1.00 [0.92; 1.08]	0.97
**Patient activation**	0.99 [0.97; 1.01]	0.21	0.99 [0.97; 1.01]	0.32
**FCR**	1.00 [0.97; 1.03]	0.99	1.00 [0.93; 1.03]	0.77

Reference group for the completion of GSD program is non-completion. GSD: Guided Self-Determination; QoL: quality of life; OR: odds ratio; CI: confidence interval; FCR: fear of cancer recurrence.

a Adjusted for age and education.

b Sheet 2.a. is excluded from the analysis.

* Statistical significance level at 5%.

*n* = 239.

**Table 4:** Associations between patient characteristics, symptoms, and total number of completed sheets among 239 patients in the MyHealth intervention group.

Characteristics	Unadjusted		Adjusted^a^	
	Beta [95% CI]	*P*	Beta [95% CI]	*P*
**Age**				
≤ 60 y	0	-	0	-
> 60 y	-1.85 [-3.10; -0.59]	0.01*	-1.89 [-3.18; -0.60]	0.01*
**Education**				
Basic or high school	0	-	0	-
Vocational training	0.31 [-1.92; 2.53]	0.79	-0.31 [-2.54; 1.93]	0.79
Higher education	-0.34 [-2.03; 1.34]	0.69	-0.88 [-2.58; 0.82]	0.31
**Cohabitation status**				
Living with a partner	0	-	0	-
Living alone	-0.61 [-1.98; 076]	0.38	-0.54 [-1.90; 0.81]	0.43
**Inclusion of a relative^b^**				
Yes	0	-	0	-
No	-3.89 [-5.09; -2.70]	<0.01*	-3.82 [-5.02; -2.62]	<0.01*
**QoL**	-0.03 [-0.07; -0.01]	0.04*	-0.03 [-0.06; 0.01]	0.13
**Symptoms of depression**	0.05 [-0.08; 0.18]	0.45	0.03 [-0.10; 0.15]	0.68
**Symptoms of anxiety**	0.106 [-0.04; 0.25]	0.15	0.09 [-0.06; 0.23]	0.24
**Patient activation**	-0.007 [-0.05; 0.04]	0.75	<-0.01 [-0.05; 0.04]	0.90
**FCR**	0.05 [-0.02; 0.11]	0.15	0.04 [-0.03; 0.10]	0.27

QoL: quality of life; CI: confidence interval; FCR: fear of cancer recurrence.

a Adjusted for age and education.

b Sheet 2.a. is excluded from the analysis.

* Statistical significance level at 5%.

*n* = 239.

### Nurses’ perspective on the GSD reflection sheets

In the focus group interview, all five nurses ranked independently the reflection sheet ‘Unfinished sentences’ as the most valued sheet in particular to initiate in-depth conversations between the nurse and patient. One nurse expressed:

*In the unfinished sentence, we are drilling deep down. (…) Then there were also some points where you could go even deeper down, and I thought that was fantastic.*

Furthermore, including a relative using the reflection sheet ‘Partner’s unfinished sentences’ was recognized by a nurse as ‘the icing on the cake’, thus highly valued as part of the program. However, the nurses addressed that some patients did not want to burden relatives with participation, while other patients either did not have a close relative or did not want them to be part of the project.

All five nurses questioned the relevance of the reflection sheet ‘Pros and cons’, two nurses questioned the relevance of the reflection sheet ‘New strategies and long-term plan for change’, while one nurse questioned the relevance of the reflection sheet ‘Bullet points you want passed on’. Furthermore, the nurses argued that certain reflection sheets might need refinement as they realized some patients found them difficult to complete, including ‘Important events and periods in your life’, ‘A picture, metaphor, or expression describing your life with cancer’, ‘Current problem-solving’, ‘Dynamic problem solving’, and ‘Symptoms to pay attention to after breast cancer treatment’.

### Nurses’ perspectives on factors influencing completion of the GSD program

The nurses explained how including a relative in a dialogue did contribute positively to the patients’ progression and motivation toward problem solving, and that these dialogues in some cases revealed difficult topics, drew attention to misunderstandings, and established mutual understandings between the patient and her relative. This was exemplified by a nurse sharing an example about a sick husband, whose presence and written reflections on the GSD reflection sheet ‘Partner’s unfinished sentences’ opened-up a dialogue on difficult topics between the patient and her husband:

*Well, he come with a walker, he smells of urine, he can hardly drag his legs with him, he has spent all his energy on coming and supporting, and almost the only thing he has written [on the refection sheet] is: I think she [the patient] should live her life, I think she should get well, I do not think she should be stuck with me.*

The nurse explained that the woman had been feeling guilty by being a burden to her husband, and that she was unaware of her husband’s readiness to support her, but that this dialogue helped her move forward.

The nurses identified three reasons for patients not completing reflection sheets. First, according to the nurses, patients who found them unnecessary did not complete them, and this also caused less progression over time causing frustrations for the nurses. Second, some patients found the content too emotional or private to talk about. One nurse described that a patient skipped a sheet expressing ‘this is hurting’. Third, some patients did not know how to approach a particular sheet. This was especially seen for elderly patients or patients with lower educations, and some patients not being familiar with reflecting on their own thoughts and emotions. Concerning socioeconomic factors as barriers, one nurse mentioned a patient having dropped out of school in seventh grade who was ‘completely overwhelmed by them [the sheets]’. However, the nurses stressed that the GSD program was still useful for the less resourceful patients because it could be applied in alternative ways, e.g. in collaboration with the nurses, who thus did the writing on the sheets on the patients’ behalf.

## Discussion

This study is the first to evaluate fidelity and acceptance of the GSD program as part of a large RCT. We found moderate-to-high fidelity: the majority of the patients completed the program, whereas 81% completed at least the short version, and the average proportion of completed reflection sheets in the short version was 71% [11]. Both quantitative and qualitative findings indicate that the values-based reflection sheet ‘Unfinished sentences’ and including a relative were driving forces for using the GSD program. Notably, patients with lower education were more likely to complete the program, while psychological symptoms did not affect patients’ completion of the program or completion of reflection sheets. These findings support the demonstrated long-term effect of the MyHealth study, thus future implementation and use of the program [10].

Compared to previous studies on psychosocial interventions for BC patients with completion rates ranging between 48 and 77% [17, 29], we do consider the completion rate of 81% to be high. A systematic review investigating fidelity in 28 psychosocial intervention studies in oncology [12] found mean adherence to interventions of 57% across studies corresponding to moderate fidelity. In our study, the nurses found the GSD program highly applicable and valuable for the BC patients, as the reflection sheets supported important dialogues and reflections. We consider that the identification of the three most frequently completed reflection sheets ‘Invitation to collaborate’, ‘Symptoms to pay attention to after breast cancer treatment’, and ‘Unfinished sentences’ was of major importance for future implementation of the GSD program and the MyHealth intervention. The first sheet ‘Invitation to collaborate’ is essential for establishing collaboration with clear boundaries and encouraging different points of view to be clarified in a constructive way [20]. The second sheet ‘Symptoms to pay attention to after breast cancer treatment’ was central as it enabled the patients to differentiate between harmless symptoms and high-risk symptoms. This was in accordance with results from a study of patients with gynecological cancer who reported that a similar sheet helped reducing FCR [7]. The third sheet ‘Unfinished sentences’ was emphasized by the nurses to be the most valuable sheet in the program and fundamental for allowing in-depth conversations by enabling patients to discover values they previously were not attentive to. Previous studies on the GSD program have identified this sheet as an appropriate starting point for in-depth communication encouraging patients to go into detail about their answers [20].

Our findings suggest the importance of including a relative to the GSD session. Including a relative may have enhanced fidelity by exchange of knowledge and emotions, which may mobilize the capacity to collaborate [30]. This is in line with a systematic review of behavioral interventions in BC, showing that social support was associated with increased adherence to hormonal therapy in BC survivors [31]. Our findings that elderly patients were less likely to complete the GSD program strengthen findings from previous studies on dropouts in psychosocial interventions [15, 16]. However, other studies examining psychotherapy and symptom assessment interventions among patients with advanced cancer found no association between age and completion [14, 18]. Further research regarding the impact of age on completing psychosocial interventions is needed.

Previous studies on psychosocial interventions showed that lower education was associated with non-adherence among cancer patients [15–17], whereas other studies examining psychotherapy and symptom assessment interventions among cancer patients found that education did not predict dropout [14, 18]. We found that lower education did not decrease the completion of the GSD program; on the contrary, patients with basic and high school were more likely to complete relative to patients with higher education. This suggests that GSD is also suitable for patients with lower socioeconomic positions. Still, the nurses indicated that they may have compensated for some of the challenges by assisting some patients in completing the reflection sheets during their conversation. Future studies could explore if the reflection sheets that nurses highlighted as difficult for some patients could be adjusted, and GSD programs should ensure that sufficient time and competencies are available for supporting patients. One may speculate that patients with higher education had lower completion due to lower need for support, but we do not have sufficient knowledge to make conclusions, and further research is needed to explore the impact of patients’ socioeconomic position on intervention completion.

Our study was subject to strengths and limitations. The conceptualization of fidelity varies across studies [12, 19, 32, 33]. The golden standard approach to evaluate intervention fidelity is to assess the actual application and use of intervention tools through audio or video recordings [32], which was not possible in the current study. However, a strength was the unique access to registrations on reflection sheets to measure the GSD program. Another strength is that participants in the MyHealth study were comparable to other low-to-moderate risk BC patient populations [34], indicating that our results are generalizable to this population. We also consider the focus group interview with the nurses a major strength due to their extensive experience with delivering the GSD program to 239 patients. The MyHealth study is the largest to evaluate the GSD program allowing quantitative analyses. In line with a previous study, possible recall bias was limited, as the nurses prior to the focus group interview had reviewed completed reflection sheets and notes from their sessions, which supported their memory of specific patients [35]. Finally, the results should be interpreted with caution as the assumptions of normality for the linear models were in some cases not met.

## Conclusion

We found that the GSD program in the MyHealth study was delivered with moderate-to-high fidelity. Patients who included a relative and patients with lower education were more likely to complete the GSD program. Furthermore, patients who included a relative and younger patients completed more reflection sheets. Finally, nurses found that the GSD program was highly applicable for the BC patients. Our findings provide specific suggestions for including the GSD program in BC care.

## Supplementary Material

Fidelity of the Guided Self-Determination program in the MyHealth study during breast cancer follow-up

## Data sharing

The data cannot be shared as the participants did not consent to this.
