# Supplementary material for: Fidelity of the Guided Self-Determination program in the MyHealth study during breast cancer follow-up
Source: Acta Oncol. 2025 Feb 17;64:42253. doi: 10.2340/1651-226X.2025.42253 (PMC11848942; doi:10.2340/1651-226X.2025.42253)
Supplement: Fidelity of the Guided Self-Determination program in the MyHealth study during breast cancer follow-up [file AO-64-42253-s1.pdf]

Supplementary material has been published as submitted. It has not been copyedited, or typeset by Acta Oncologica

Supplemental table 1: Purpose of GSD reflection sheets

Patterns to be changed	Session 1	Purpose
Many women meet with reduced expectations of professionals and have adapted a disease-oriented approach and a passive patient role. The women have rarely set conscious goals for the interaction.	**Invitation to collaborate** 1.a	Used to expand the woman's expectations and to prepared them to take an active role focusing on what they currently fin difficult living with the disease.
Problematic conditions or events earlier in the woman's life can disrupt and prevent the current management of the disease. Although nurses spend a lot of time getting the woman to talk about their life with the disease, significant points can be lost.	**Important events and periods in your life** 1.b	The woman's story is maintained to provide an overview and promote her identity. The intention is to direct the woman's attention to herself in the past, present, and future. The woman is reminded that the current process is an election and that the elections may have an impact on the future. When life events are made visible, the woman can discover connections she has not seen before
Often the nurse focuses on some standard problems and the doctor on the disease-specific. Hereby, the woman is not able to tell what is difficult to her. Other times, they spend a lot of time talking about something that is not relevant.	**What do you find difficult at present?** 1.c	Directs the woman's attention to current issues. Invite her to talk about the things that are difficult, in her own words.

Often, women have not recognized how different aspects of their everyday life can be related to difficulties in dealing with their illness. This can be painful or embarrassing for the women to talk about or just think about. Sometimes they form "blind spots". Women rarely think that professionals find these matters relevant.	**Unfinished sentences**<br>1.d.1 + 2	The sentences can help the woman to discover and talk about difficult topics, to gain new knowledge, and to become aware of what is important to her. Provides quick information on many topics that can make them easier to talk about.
Many women spontaneously describe their situation using metaphors or automatic thoughts, which often reveal their overall perception of living with the disease. If metaphors and automatic thoughts remain unchallenged, they can maintain the perception whether it is positive or negative, even if it is so negative that it omits all hope.	**A picture, metaphor, or expression describing your life**<br>1.e	When the woman's spontaneous metaphors or automatic thoughts about the disease are discovered it can be seen as a gift from deeper and hard-to-reach layers of consciousness that "reveal" the basic attitude to the disease. In this way, a negative attitude can become the subject of communication and thereby be challenged and modified.
The problems in the period after cancer are diverse and many are difficult to get started to talk about.	**Current experiences on difficulties and nuisances**<br>1.f	Gives both the woman herself and the nurse a quick overview of the woman's own problems and their duration. Puts difficult areas on the agenda and makes them easier to talk about.
**Session 2**		
The woman and her partner/relative may avoid talking about their mutual thoughts on difficult topics related to the disease and the treatment. It can	**Partner's unfinished sentences**<br>2.a	The unfinished sentences make room for the relative's thoughts and help the woman and the relative to discover and talk about important

make it harder for them to support each other and help the woman further in life. The partner or another important person can become disconnected and important resources can be lost in the relationship.		topics. Often this can “break the ice” and make it easier for the parties to talk about the topics afterwards. Should more difficult conflicts become apparent, the need to refer to a psychologist may be considered
The professionals find it difficult to get an overview of how the woman copes with living with her new situation as a cancer survivor and how she acts on the challenges it presents. Both time, energy and effort are often used without giving an overall insight into the specific person’s readiness to change specific areas.	**My daily life and needs for changes** 2.b	Provides a quick overview of the woman’s needs and potential for change. The woman can openly propose something that could be changed, without deciding to change it. It signals respect for the woman’s choice and stimulates the woman to take an independent position. The sheet is used to select the areas that are relevant to work with.
For some women, the disease fills so much that one can say that it forms a significant part of their problems. However, this is a form of reaction that professionals rarely talk about. Likewise, it seems to be difficult to approach the opposite issue, where women try to neglect their disease.	**Room for the fact, that you have been treated for cancer** 2.c	The sheet asks for the woman’s own assessment of whether she is giving her illness appropriate, too little, or too much space. It is expected that the woman’s assessment will often correspond to the professional. Whether this is the case or not, the woman’s proposal can be used in a constructive discussion about how and how much the disease should fill in her life. Enables the woman to distinguish between negative and positive ways the disease can fill. Provides a chance to actively

		determine and put into words an overall change in how and how much the disease should fill. Can help the woman to reach and express the first acceptance of the disease.
**Session 3**		
Patient and professional rarely know each other's experiences of what they each find important in the woman's situation. They rarely get to make a joint decision about what they should work with - or not work with.	**Current problem-solving**<br>3.a	The woman's and the nurse's lists of what they find important to focus on are compared. They must be different. Inspired by the lists, they jointly formulate a problem or challenge brief and precisely. The problem is described in a way that both patient and nurse perceive as real and comprehensive. It is given the following form:<br>*"What I find difficult, or challenging is…"*
In a conversation with professionals, patients may play a passive role	**Your draft**<br>3.a.1	The sheet gives the woman the opportunity to independently make status and formulate as well as express her own thoughts
Nurses may also find it difficult to express views that she does not believe the patient shares.	**The nurse's draft**<br>3.a.2	Helps the nurse to endure the tension that arises when different perceptions meet. It can contribute positively to change.
**Session 4**		
Professionals have very little knowledge about the woman's problem solving so far. Often, 'gaps' or inconsistencies in	**Problem solving so far**<br>4.a.1 - 4	In the area, they choose to work with, the nurse must know the woman's previous problem solving.

the woman's previous problem solving cause it not to be effective. Rarely do they get agreed or named which problem areas they want to work with or not work with.		A detailed review of one's own problem solving can strengthen the woman's decision-making ability and focus and help her to distinguish between consideration for herself and others and between short- and long-term interests.
Professionals have often had a fragmentary view of the woman's life. But a detailed picture is necessary if the professional is to be able to support the woman in an improved problem solving.	**Dynamic problem solving**<br>4.b	Further knowledge about the woman's observations, thoughts, goals or actions is made visible in the joint reflection on the woman's previous problem solving. It leads to the development of judgment in the woman.
**Session 5**		
Treatment for breast cancer can lead to increased awareness and concern about even the smallest symptoms from the body. It can be difficult to distinguish between symptoms that the cancer has returned, following treatment or regrettable symptoms that are related to everyday life.<br><br>It is important to know that not all signs from the body are signs that the cancer has returned.<br><br>The surgery and radiotherapy can have a number of consequences for your	**Symptoms to pay attention to after breast cancer treatment**<br>5.a	The sheet gives a listed a number of symptoms that may be a sign that the cancer has returned. It is important that the woman react to these symptoms if they persist and affect the everyday life.<br><br>Woman should react if she notices any of the symptoms below.<br>• New nodule either in the breast area or in the lymph nodes<br>• Pain in the bones, back, hip or elsewhere

body's appearance and function, including:<br><br>• Pain and reduced mobility in the shoulder and arm<br>• Sensory disturbances<br>• Swelling of the arm, also called lymphedema,<br>• Scar tissue formation<br>• Skin changes<br>• Altered appearance of cosmetic genes		• Pressure across the upper abdomen<br>• Extreme fatigue<br>• Difficulty breathing and coughing<br>• Sudden unintentional weight loss<br>• Nausea
At the end of the program, the change work is still in progress. New realizations, goals and actions may have been initiated, but not firmly rooted in everyday life. Overall goals of change can be difficult to pursue.	**New strategies and long-term plan for change**<br>5.b	The sheet is used to take stock of what has been achieved as well as see the significance of it in the future. Can help the woman to implement and obtain support to sustain change in the long term. Maintains an overall goal over time and provides space to clarify short-term goals and efforts.
The woman may be in a dilemma and have difficulty figuring out the pros or cons of a choice. In this way, her opposing interests may block decision-making.	**Pros and cons**<br>5.c	The sheet helps the woman to clarify the advantages and disadvantages of a reaction. This can stimulate decision-making so the woman with herself knows why she will change or not change behavior.
Some of the challenges the woman has been working on are lengthy and continuing working on them can be	**Bullet points you want passed on**<br>5.d	The note here can be used as a kind of to-do list. It could, for example. be to a relative or the doctor.

supported by others, which can be difficult to initiate in the case.		
